# Supplementary material for: Polyzwitterion–SiO2 Double-Network Polymer Electrolyte with High Strength and High Ionic Conductivity
Source: Polymers (Basel). 2023 Jan 16;15(2):466. doi: 10.3390/polym15020466 (PMC9861860; doi:10.3390/polym15020466)
Supplement: Supplementary file 1 [file polymers-15-00466-s001.zip › polymers-2126142-supplementary.pdf]

Supporting Information

Polyzwitterion-SiO_2 Double Network Polymer Electrolyte with High Strength and High Ionic Conductivity

Lei Zhang,[a] Haiqi Gao,[b] Lixiang Guan [d] Yuchao Li [c]* and Qian Wang[d]**

[a]*School of Materials and Chemical Engineering, Chuzhou University, 1528 Fengle Avenue, Chuzhou 239099, China.*

[b]*State Key Laboratory of Chemistry and Utilization of Carbon Based Energy Resources; College of Chemistry, Xinjiang University, Urumqi 830017, Xinjiang, PR China.*

[c]*School of Materials Science and Engineering, Liaocheng University, 252000, PR China.*

[d]*Institute of Energy Innovation, College of Materials Science and Engineering, Taiyuan University of Technology, Taiyuan 030024, China.*

E-mail: qianwang19930825@163.com; *liyuchao@lcu.edu.cn;* iamhqgao@njupt.edu.cn

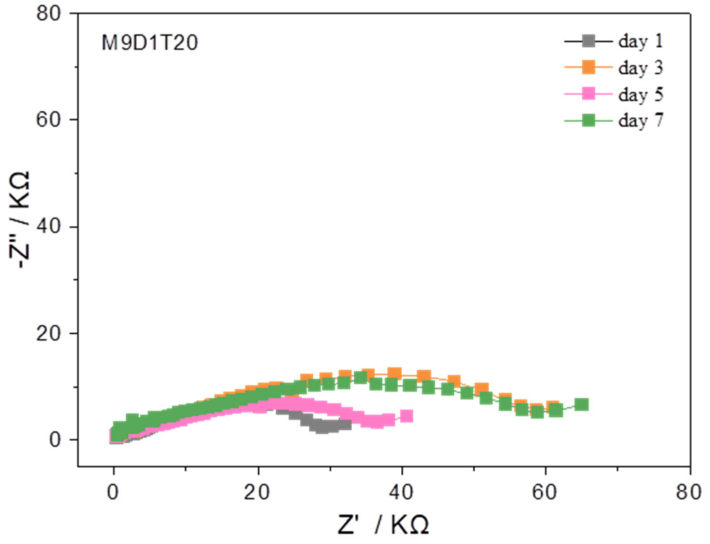

Figure S1. EIS of Li/Li cell using M9D1T20.

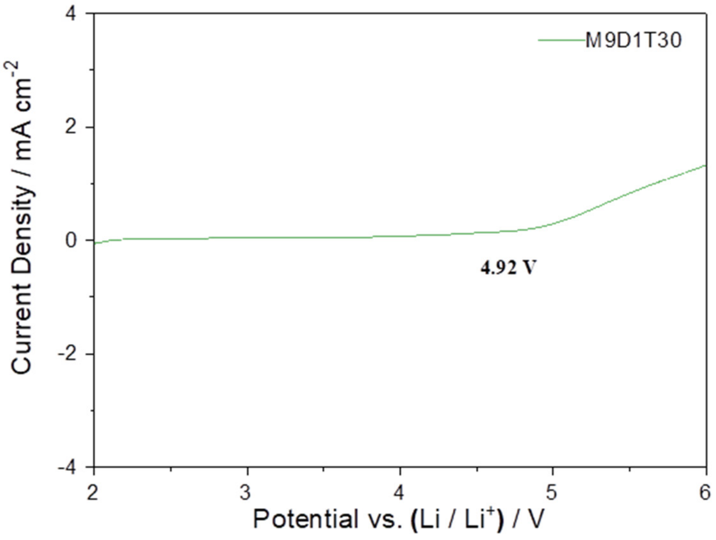

Figure S2. Electrochemical stability of M9D1T30

Table S1. Weight percentage of each component of MxDyTz, the strength, stress and ionic conductivity also are shown.

MxDyTz	W_{MPC} (%)	W_{DPS} (%)	W_{TEOS} (%)	Stress/ %	Strain/ Mpa	Ionic conductivity / mS cm^{-1} (30 °C)
M5D5T20	5	5	20	0.3	195	0.16
M10D0T20	10	0	20	0.45	367	0.44
M9D1T15	9	1	15	0.24	419	\
M9D1T20	9	1	20	0.55	439	0.31
M9D1T25	9	1	25	0.27	560	\
M9D1T30	9	1	30	0.75	569	0.3
M9D1T35	9	1	35	0.28	550	\
